# Exploring the Impact of Open Pedagogy on Minority Students’ Motivation, Computational Thinking, and Perceived Learning in Interactive Computer Game Development

**DOI:** 10.3390/jintelligence14010016

**Published:** 2026-01-19

**Authors:** Yu-Tung Kuo, Yu-Chun Kuo

**Affiliations:** 1 Department of Applied Engineering Technology, North Carolina A&T State University, Greensboro, NC 27401, USA; 2 Department of Critical Literacy, Technology & Multilingual Education, Rowan University, Glassboro, NJ 08028, USA; kuo@rowan.edu

**Keywords:** open educational resources (OER), open pedagogy, motivation, computational thinking, computer programming, computer game development

## Abstract

The use of open educational resources (OERs) is on the rise in higher education. Open pedagogy, as a learner-centered approach, provides students with opportunities to create, design, or adapt openly licensed materials or resources. With the potential of open pedagogy to enhance student learning, this study investigated the effect of an open pedagogy project on minority students’ motivation and perceived learning in the computer game programming course. An experimental design was implemented to compare minority students’ learning in programming through the open pedagogy approach versus the traditional approach. Participants were fifty-eight minority students enrolled in game courses from an institution in the southeastern United States. Thirty students received the instruction with open pedagogy, while twenty-eight students were in the traditional instruction. Quantitative approaches were performed to analyze the collected data. The results indicated that minority students in the open pedagogy group perceived significantly higher levels of motivation on the aspect of pressure/tension than those receiving the traditional approach. Minority students participating in the open pedagogy project had significantly higher levels of computational thinking and perceived learning performance in computer programming, compared to the students with the traditional instruction. Major findings and limitations of this study (i.e., short intervention period, small sample size, etc.) were reported and discussed.

## 1. Introduction

The open education movement has led to increasing interest from educators, advocates, and learners in the use of open education resources (OERs) and the application of open pedagogy in different disciplines (Clinton-Lisell & Kelly, 2024; Y. C. Kuo & Kuo, 2025; Short et al., 2024). Open pedagogy supports student learning by providing students with opportunities to engage in the process of creating and editing open educational resources (OERs), through which students learn the new concept or content (Clinton-Lisell, 2021; Clinton-Lisell & Kelly, 2024; Lazzara et al., 2024). Students often face challenges when learning computer programming, especially for minority students who are usually less motivated or persistent, which leads to high chances of failure rates in courses relevant to programming (Cheah, 2020; Y. T. Kuo & Kuo, 2023; Ober et al., 2024).

Open pedagogy, with its emphasis in learner-centered learning and the benefits in facilitating active learning (Casey et al., 2022; Lazzara et al., 2024; Wiley & Hilton, 2018), may have the potential to improve students’ motivation to learn programming and learning performance in programming knowledge and skills. There is a lack of empirical studies on open pedagogy in minority-focused programming education (i.e., African Americans, etc.). Existing research on open pedagogy was mostly carried out in the social science fields (e.g., psychology, education, etc.), and there is limited open pedagogy research in STEM, but none was conducted in computer programming (Biddle & Clinton-Lisell, 2023; Clinton-Lisell & Gwozdz, 2023; Rollag Yoon & Gilpin, 2022; Williams, 2022). Limited OER studies were conducted in the field of computer programming education, and they primarily focused on the mere use of OERs (e.g., software, educational games, etc.) in computer programming classes (Da Silva et al., 2020; de Deus et al., 2024; Da Silva & Silveira, 2020), not on the creation or design of OERs based on open pedagogy.

The effectiveness of the adoption of OP in facilitating the development of minority students’ attitudes, knowledge, and skills in computer programming is unclear. In addition, to ensure the benefits of adopting open pedagogy for students learning computer programming, empirical studies with an experimental design with comparison groups are needed to verify the impact of open pedagogy on student learning (Clinton-Lisell & Kelly, 2024), and there is a lack of such OP studies for minority students. Therefore, this study aimed to design an OER creation activity as project-based open pedagogy, and examined its effect on minority students’ motivation, computational thinking, and performance in learning computer programming. Three research questions were proposed as follows:

1. What are the differences in minority students’ motivation to learn programming between the OP group and the non-OP group?
2. What are the differences in minority students’ computational thinking between the OP group and the non-OP group?
3. What are the differences in minority students’ perceived learning performance in computer programming between the OP group and the non-OP group?

## 2. Literature Review

This section includes a review of the literature on open pedagogy, computer programming, and other relevant concepts covered in this study.

### 2.1. Open Educational Resources

According to UNESCO, open educational resources (OERs) are “learning, teaching and research materials in any format and medium that reside in the public domain or are under copyright that have been released under an open license, that permit no-cost access, re-use, re-purpose, adaptation and redistribution by others” (UNESCO, 2019, para. 19). OERs can include “full courses, course materials, modules, textbooks, streaming videos, tests, software, and any other tools, materials, or techniques used to support access to knowledge” (Hewlett, 2016, para. 7). Wiley and Hilton (2018) described the “Five R (5Rs) Permissions” of OER as Retain, Reuse, Revise, Remix, and Redistribute. OERs provide a huge potential for educators to include these materials in their courses in various ways, helping reduce learning costs and improve learning quality and ensuring inclusive education (Bliss & Smith, 2017; Clinton-Lisell, 2024; Fischer et al., 2015; Hilton et al., 2019; Y. C. Kuo et al., 2024; Zhang et al., 2020).

### 2.2. Open Pedagogy

Open pedagogy (OP) is a term with a long history and has been used in various contexts with varied interpretations (Wiley & Hilton, 2018). With an emphasis on students’ needs and preferences in a learning context, open pedagogy is student-centered and allows students to co-create the context of the class (Daniel, 2004; Wiley & Hilton, 2018). In open education with the use of OERs, open pedagogy refers to the creation and editing of open educational resources (OERs) to support learning (Clinton-Lisell, 2021; Clinton-Lisell & Kelly, 2024; Lazzara et al., 2024). Clinton-Lisell and Kelly (2024) indicated open pedagogy as an approach centering on “students creating and editing openly licensed materials for others to use” (p. 568). DeRosa and Robison (2017) described open pedagogy “access-oriented commitment to learner-driven education AND as a process of designing architectures and using tools for learning that enable students to shape the public knowledge commons of which they are a part” (pp. 13–14). Open educational practices (OEPs), another term emerging from the use of OERs, share some similar concepts or techniques with open pedagogy, and they involve the use, creation, or reuse of OERs (Clinton-Lisell, 2021; Wiley & Hilton, 2018). Although with an overlap between open pedagogy and OEP, open pedagogy goes beyond the simple use of OERs under OEP, and focuses on using OERs in a more complex way that involves creating or editing OERs (Clinton-Lisell, 2021; Paskevicius & Irvine, 2019).

Open pedagogy is associated with several theories or pedagogical approaches, including active learning, project-based learning, collaborative learning, experiential learning, or peer learning, as they are all student-centered learning approaches and share the same perspective in constructivism (Casey et al., 2022; Wiley & Hilton, 2018). Depending on the design of OER activities, open pedagogy can be implemented to support other pedagogical approaches (e.g., inquiry-based learning, project-based learning, flipped learning, etc.), or vice versa (Casey et al., 2022; Rollag Yoon & Gilpin, 2022; Williams, 2022). With open licensing and the 5Rs, open pedagogy provides students with opportunities to participate in a wide range of activities (e.g., making copies, developing derivative materials, etc.) that may not be achieved through the use of copyrighted materials (Wiley & Hilton, 2018). It empowers students to engage in the knowledge creation or construction process, through which students work collaboratively and contribute to the development of new knowledge, concepts, or skills (Bali et al., 2020; Lazzara et al., 2024).

With open pedagogy, students are not passive knowledge consumers; instead, they become active knowledge creators (Clinton-Lisell, 2021). Open pedagogy assignments or projects, involving the adaptation or creation of openly licensed materials or resources, can create a learning environment that promotes student-centered learning in which students shape their own knowledge or learning experience through collaborative knowledge construction (Casey et al., 2022). The processes of co-creation or co-editing allow students to voice their opinions, which in turn facilitates diversity, inclusion, and accessibility of learning materials or resources (Casey et al., 2022; Clinton-Lisell, 2021; Zhang et al., 2020). Examples of open pedagogy assignments or projects include, but are not limited to, creating websites or visuals, editing OER content, story books, or Wikipedia articles, co-developing audio or videos describing a new concept or demonstrating examples, developing learning resources for others (Clinton-Lisell, 2021; McKenney et al., 2024; Short et al., 2024; Werth & Williams, 2021).

### 2.3. Benefits of Open Pedagogy in Learning

Open pedagogy has been deemed to have a positive influence on students’ learning experience and outcomes, such as motivation, learning performance, skill development, etc. (Clinton-Lisell, 2021; Clinton-Lisell & Gwozdz, 2023; Short et al., 2024; Trust et al., 2023). Clinton-Lisell (2021) conducted a systematic review of empirical findings on open pedagogy, and found that students’ experiences with open pedagogy are generally positive. With open pedagogy, students’ roles shift from knowledge consumers to the curators and designers of OERs, leading to an increased motivation, positive attitudes towards learning, and the development of 21st century skills (Trust et al., 2023). Open pedagogy projects with students as the creators and designers foster learner agency that is critical to the internalization of student motivation, and create a learning environment that is student-empowered and student-centered (Werth & Williams, 2021).

Compared to traditional assignments, open pedagogy assignments lead to higher levels of students’ motivation to learn (Clinton-Lisell & Gwozdz, 2023; Lazzara et al., 2024). Students engaged in renewable assignments reported higher levels of intrinsic motivation in interest/enjoyment, pressure, perceived choice, and perceived competence, than those working on traditional assignments (Clinton-Lisell & Gwozdz, 2023). The use of renewable assignments in open pedagogy provides diverse learning opportunities, leading to learning benefits such as improved motivation and interest, meaningful knowledge construction and exchange, course content learning, skill development, and personal growth (Lazzara et al., 2024).

The open pedagogy approach can enhance learning outcomes, such as mastery of core academic content, skills in collaborative learning, critical thinking and problem solving, effective communication, and learning how to learn (Mayer, 2023; Short et al., 2024). From instructors’ viewpoints, increasing student performance is one of the perceived affordances of open pedagogy, as open pedagogy makes learning more visible through students’ openly display of their learning or their conversations with those interested in their work (Short et al., 2024). In a meta-analysis of open educational resources and practices, OERs were found to have a statistically significant effect on learning achievement, with varying impacts based on course subjects, educational levels, and geographical contexts (Tlili et al., 2023).

### 2.4. Computer Programming and Computational Thinking

Computer programming has been included in computer literacy education in many countries in the last decade (Tsai et al., 2021). Exploring effective approaches or instructional strategies to enhance students’ motivation and experiences in learning coding or computer programming has been a prevalent topic in computer science (CS), CS education, educational technology, STEM, or relevant fields (Husain, 2024; Y. T. Kuo & Kuo, 2025; Mellado & Cubillos, 2024; Su & Yang, 2023; Tsai et al., 2021; Zhang et al., 2024). In particular, minority students often present low motivation and learning performance in computer programming (Y. T. Kuo & Kuo, 2023; Ober et al., 2024).

Computational thinking (CT), referring to the mental processes or fundamental skills rooted in computer science for problem solving, is critical to the success of computer programming learning (Tsai et al., 2021; Wing, 2006). Conceptually, CT has been described as a multidimensional construct comprising computational concepts, practices, and perspectives (Brennan & Resnick, 2012; Cansu, 2019), and is enacted through key cognitive processes such as decomposition, abstraction, pattern recognition, and algorithmic thinking (Wing, 2006; Yadav et al., 2016). CT is intrinsically associated with computer programming, through which learners routinely engage in practices such as looping, conditional logic, event handling, debugging, etc. (Atmatzidou & Demetriadis, 2016; Brennan & Resnick, 2012; Jeon & Kwon, 2024; Zhou et al., 2024). Consequently, programming education serves as an important avenue for fostering CT development, with engagement in programming activities shown to enhance learners’ higher-order cognitive and computational thinking skills (Tsarava et al., 2022; Zhou et al., 2024).

## 3. Method

This section describes the methodological procedures employed in the study, including the sample of the participants, the data collection process, and the details of the context and design of this study.

### 3.1. Participants

The participants in this study were 58 minority students enrolled in an undergraduate course of Game Development. The course was offered face-to-face in the College of Science and Technology at an HBCU (Historically black college and university) in the southeastern United States. Among the 58 students, there were 28 students in the non-OP group and 30 students in the OP group (see Table 1). The two groups were enrolled in the Game Development courses in Fall and Spring semesters, respectively. In the non-OP group, there were 15 males and 13 females. All of the students were between 20 and 23 years old, except for one student who was older than 23. In the OP group, half of the students were male students, and the other half were female students, aged from 19 to 23 years old. In both non-OP and OP groups, most of the students were juniors (83.3%) and some were seniors (16.7%). The non-OP and OP groups, respectively, included 100% and 96.7% African American students.

### 3.2. Data Collection

Data were collected using an online survey. The study was approved by the university’s Institutional Review Board (IRB), and informed consent forms were obtained from the students who participated in the survey. The online survey, delivered through the university’s survey platform, was provided to minority students at the end of the open pedagogy project. The survey questionnaire consisted of four sections, including student background information, motivation to learn programming, computational thinking, and perceived learning outcomes in computer programming (see Table 2). Student background information consisted of gender, age, ethnicity, and grade levels.

Table 2 provides an overview of the scales used in the study. The motivation survey was used to investigate the learner’s desire to engage in learning computer programming. This survey was adapted from the Intrinsic Motivation Inventory (IMI) created by Deci and Ryan (2000) and includes interest/enjoyment, perceived competence, effort/importance, pressure/tension, perceived choice, and value/usefulness. The wording of “the activity” was changed to “computer programming” for this study. The scale was a 7-point Likert scale ranging from 1 (strongly disagree) to 7 (strongly agree). The Computational Thinking survey was applied to examine the learner’s computational thinking processes in a computer programming activity. This survey was adopted from the Computational Thinking Scale (CTS) developed by Tsai et al. (2021). It includes abstraction, decomposition, algorithmic thinking, evaluation and generalization sub-scales. The survey was a 5-point Likert scale ranging from 1 (strongly disagree) to 5 (strongly agree). The survey of perceived learning outcomes in computer programming, adopted from the survey developed by Y. T. Kuo et al. (2025), was used to measure students’ self-evaluation regarding the knowledge and skills of computer programming learned in the class. This survey includes Understanding, Application, and Problem-Solving subscales. The scale was a 5-point Likert scale ranging from 1 (strongly disagree) to 5 (strongly agree). The values of Cronbach’s Alpha for the above scales in this study were listed in Table 2. The values of the Cronbach’s alpha of all subscales were above 0.8 or 0.9, indicating good or excellent reliability (George & Mallery, 2020).

### 3.3. Context and Research Design

Students enrolled in the game development course were required to use OERs to design and develop OER materials to teach programming to beginning learners. This OER project took three weeks to complete. Before the project began, the instructor taught students the concept of OERs and shared relevant OER platforms, examples, and resources. Major tasks for students in this project included (1) designing the practice computer game coding examples, (2) developing OER instructional materials including the user manual (lecture slides), demo videos, and coding scripts, and (3) applying one of the Creative Commons licenses to the materials they developed.

The nonequivalent control group posttest-only design was conducted in this study. The control group includes the undergraduate students (*n* = 28) enrolled in the Fall computer game programming course; the experimental group includes the undergraduate students (*n* = 30) enrolled in the Spring computer game programming course. The computer game courses were taught by the same instructor. The experiment period was 3 weeks, including the computer programming lecture (1 week) and the assignment lab time (2 weeks). The total time was around 7.5 h. In the game course, the students use the Unity, a cross-platform game engine as the primary software to develop computer games. Specifically, in the beginning of the lecture, both groups were asked to complete the online background survey that includes information on students’ majors, whether they took the web design I course, and levels of programming skills (i.e., basic vs. high). All students reported having taken the web design I course, and rated their programming skill at the basic level. All students were in the same major (computer graphics technology), except for one student who majored in computer system technology who was removed from the data analysis. Next, the instructor provided the lectures and demonstrations (via Unity) for the computer programming topics, including Variables, Operators, Control Flows (i.e., if/else, for, while loops), Inputs, and Methods. After the lectures and demos, the students in the control group were assigned the computer programming assignments designed based on the traditional assignment mode. On the contrary, the students in the experimental group were assigned the computer programming assignments designed based on the open pedagogy mode. After the student submitted their assignments, both groups were asked to complete the Motivation Survey, Computational Thinking Survey, and the survey of perceived learning outcomes in computer programming.

**Figure 1:**
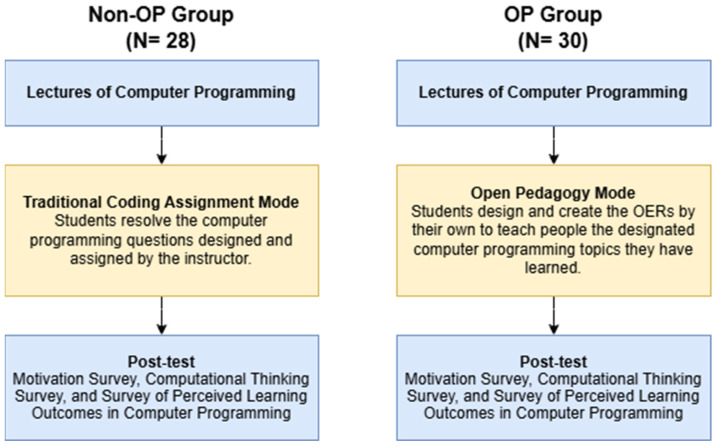
Design of the Study.

The design of control and experimental groups (see Figure 1):

A. Control Group (Non-OP Group): The students were asked to implement the game programming questions assigned by the instructor. These questions were designed based on the learning content including the use of Variables, Operators, Control Flows (i.e., if/else, for, while loops), Inputs, and Methods, respectively. This is individual work and each student needed to script and implement the code in Unity to provide the solution for each question designated by the instructor. This is the common traditional mode of assignment in a computer programming course.

B. Experimental Group (OP Group): The students were asked to design and create the Open Educational Resources for the topics they have learned in the class. Specifically, the students in the OP group were placed into a scenario in which they played a role as an instructor and creator. They were asked to create the instructional materials to teach computer programming beginners to learn the content including the use of Variables, Operators, Control Flows (i.e., if/else, for, while loops), Inputs, and Methods (the scope is the same as the control group). Before the assignment started, the instructor provided a short 15 min lecture to introduce the Open Education Resources and the Common Licenses to allow the students to understand the purposes of the assignment. In the assignment, the students needed to: (1) plan the instructional content, (2) design and implement their own code scripting examples in Unity, and (3) create their instructional slides and videos. The students were required to apply one of the Creative Commons licenses to the materials they developed.

### 3.4. Data Analysis

The data were analyzed by applying quantitative approaches. Quantitative approaches consisted of descriptive and *t*-test analyses. SPSS 26 was used for data analyses. The sample sizes for the control and experimental groups were adequate for *t*-tests, as King and Eckersley (2019) indicated that student’s *t*-tests were widely used when the sample size is reasonably small, less than approximately 30.

## 4. Results

### 4.1. RQ1: What Are the Differences in Minority Students’ Motivation to Learn Programming Between the OP Group and the Non-OP Group?

**Figure 2:**
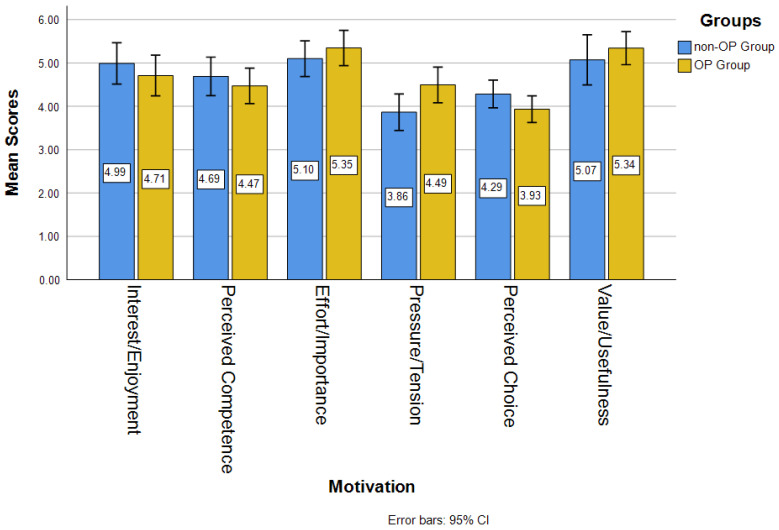
Mean Scores of Motivation.

Figure 2 shows the mean scores of motivation in OP and non-OP groups. The OP group had higher averages in effort/importance (*M_op_* = 5.35, *M_non-op_* = 5.10), pressure/tension (*M_op_* = 4.49, *M_non-op_* = 3.86), and value/usefulness (*M_op_* = 5.34, *M_non-op_* = 5.07) than the non-OP group. The non-OP group had higher averages in interest/enjoyment (*M_op_* = 4.71, *M_non-op_* = 4.99), perceived competence (*M_op_* = 4.47, *M_non-op_* = 4.69), and perceived choice (*M_op_* = 4.29, *M_non-op_* = 3.93) than the non-OP group.

*t*-test analyses (see Table 3) show whether minority students’ motivation to learn was significantly different between the two groups. There was significant difference in pressure/tension (*t* = −2.188, *p* < .05). Minority students’ pressure/tension (*t* = −2.188, *p* < .05) was significantly higher in the OP group (*M* = 4.49, *SD* = 1.09) than the traditional group (*M* = 3.86, *SD* = 1.09). However, no significant differences were found in interest/enjoyment (*t* = 0.183, *p* > .05), perceived competence (*t* = 0.262, *p* > .05), effort/importance (*t* = −.872, *p* > .05), perceived choice (*t* = 1.632, *p* > .05), and value/usefulness (*t* = −0.809, *p* > .05) between the OP and non-OP groups.

### 4.2. RQ2: What Are the Differences in Minority Students’ Computational Thinking Between the OP Group and the Non-OP Group?

**Figure 3:**
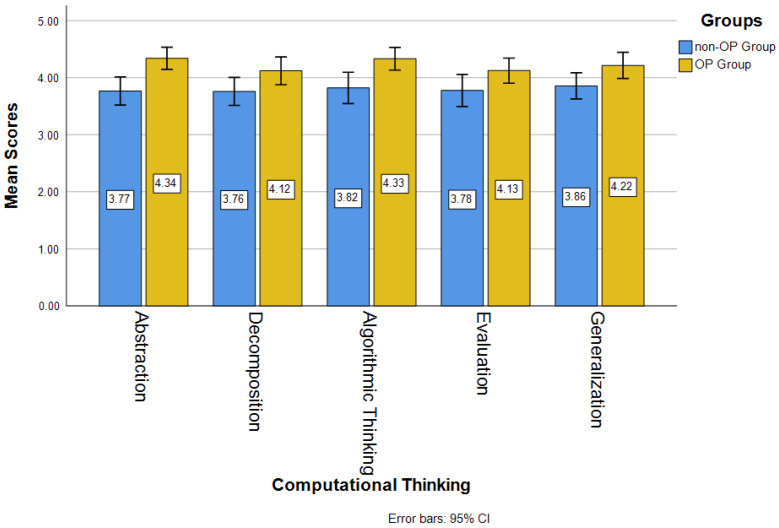
Mean Scores of Computational Thinking.

Figure 3 shows the mean scores of computational thinking in five aspects for both OP and non-OP groups. The mean scores of the OP groups in all five aspects of computational thinking, including abstraction (*M_op_* = 4.34, *M_non-op_* = 3.77), decomposition (*M_op_* = 4.12, *M_non-op_* = 3.76), algorithmic thinking (*M_op_* = 4.33, *M_non-op_* = 3.82), evaluation (*M_op_* = 4.13, *M_non-op_* = 3.78), and generalization (*M_op_* = 4.22, *M_non-op_* = 3.86) were larger than those of the non-OP groups. The mean scores of the OP group were above 4 while the mean scores of the non-OP group were below 4.

Based on the *t*-test analyses (see Table 4), the OP group had significantly higher averages in all aspects of computational thinking than the non-OP group, including abstraction (*t* = −3.769, *p* < .001), decomposition (*t* = −2.131, *p* < .05), algorithmic thinking (*t* = −3.115, *p* < .05), evaluation (*t* = −2.008, *p* < .05), and generalization (*t* = −2.267, *p* < .05). Minority students in the OP group had significantly higher levels of computational thinking in abstraction (*M_op_* = 4.34, *SD_op_* = 0.52; *M_non-op_* = 3.77, *SD_non-op_* = 0.63), decomposition (*M_op_* = 4.12, *SD_op_* = 0.65; *M_non-op_* = 3.76, *SD_non-op_* = 0.63), algorithmic thinking (*M_op_* = 4.33, *SD_op_* = 0.53; *M_non-op_* = 3.82, *SD_non-op_* = 0.70), evaluation (*M_op_* = 4.13, *SD_op_* = 0.58; *M_non-op_* = 3.78, *SD_non-op_* = 0.72), and generalization (*M_op_* = 4.22, *SD_op_* = 0.61; *M_non-op_* = 3.86, *SD_non-op_* = 0.59), compared to those in the non-OP group.

### 4.3. RQ3: What Are the Differences in Minority Students’ Perceived Learning Performance in Computer Programming Between the OP Group and the Non-OP Group?

**Figure 4:**
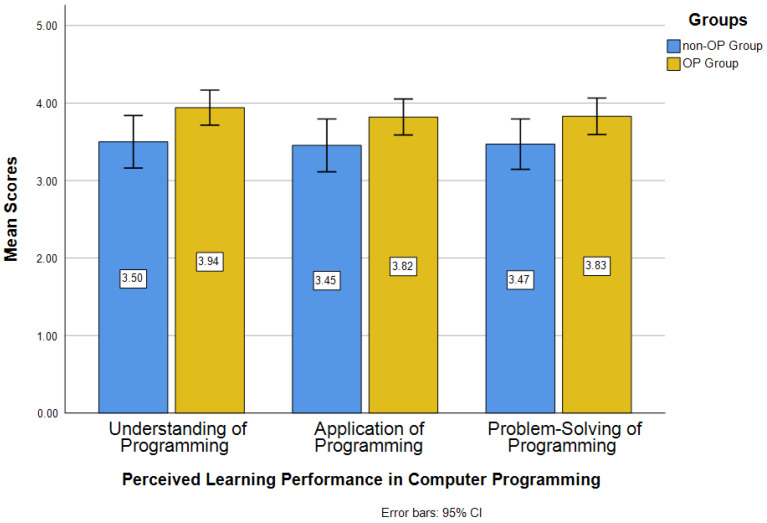
Mean Scores of Perceived Learning Performance in Computer Programming.

Figure 4 shows the mean scores of two groups in performance of computer programming. The averages of the OP group are higher than those of the non-OP group in understanding (*M_op_* = 3.94, *M_non-op_* = 3.50), application (*M_op_* = 3.82, *M_non-op_* = 3.45), and problem-solving (*M_op_* = 3.83, *M_non-op_* = 3.47) of programming. The averages of the OP group range between 3.8 and 4.0, while the averages of the non-OP group fall between 3.0 and 3.5.

Table 5 shows the results of *t*-test analyses for minority students’ perceived learning performance in computer programming between the OP group and non-OP group. The minority students in the OP group (*M* = 3.94, *SD* = 0.60) had a significantly higher level of understanding of programming (*t* = −2.241, *p* < .05), compared to the students in the non-OP group (*M* = 3.50, *SD* = 0.87). Although the OP group (*M* = 3.82, *SD* = 0.62) had a higher average in application of programming than the non-OP group (*M* = 3.45, *SD* = 0.87), but the difference of averages from two groups was not significant (*t* = −1.834, *p* > .05). Similarly, the OP group (*M* = 3.83, *SD* = 0.63) did not perform significantly better than the non-OP group (*M* = 3.47, *SD* = 0.83) in problem-solving of programming (*t* = −1.843, *p* > .05), although the mean score of the OP group was higher than that of the non-OP group.

Overall, the OP group showed significantly higher pressure/tension in motivation than the non-OP group. The OP group exhibited greater computational thinking in all aspects than the non-OP group in a significant way. For the programming performance, the OP group showed a significantly better understanding of programming, compared to the non-OP group.

## 5. Discussion

In the discussion section, we discussed the major findings of this study and explore possible causes that lead to the results. We also related the major findings of this study to those of other relevant research with similar or different findings, or the concepts that address or support the results in this study.

In terms of motivation to learn coding/programming, the OP group was found to have a significantly higher average score in pressure/tension than the non-OP group, which conforms to the claims of existing research about the potential of open pedagogy to improve student motivation to learn course content (Clinton-Lisell, 2021; Trust et al., 2023; Werth & Williams, 2021). This result implies that minority students who participated in the sessions with the use of OP possessed significantly higher levels of pressure or tension, compared to the students in the sessions delivered using the traditional lecture method. This result is contrary to the finding of the study of Clinton-Lisell and Gwozdz (2023) where students working on renewable assignments showed significantly lower pressure/tension in motivation than those working on a traditional assignment. However, the result of this study makes sense because in addition to learning coding/programming, the minority students in the OP group were asked to learn the concept of OER and use OER to create free educational materials for users to learn coding or programming, which might lead to more of the feelings of pressure or tension in motivation among minority students. These speculations are supported by the previous studies showing that highly demanding or complex tasks integrated in the learning process (such as creating instructional materials) could increase cognitive load and performance pressure alongside potential learning benefits (Anyichie & Butler, 2023; Fiorella & Mayer, 2013, 2014; Kobayashi, 2023).

As for the other five dimensions of motivation, including interest/enjoyment, perceived competence, effort/importance, perceived choice, and value/usefulness, minority students in the OP group appeared to have higher average scores in some dimensions (e.g., effort/importance and value/usefulness) and lower averages in some dimensions (e.g., interest/enjoyment, perceived competence, and perceived choice), compared to those in the non-OP group. However, the differences were not significant between the two groups of minority students, the OP or non-OP groups. The results indicated that minority students’ motivation levels in learning coding did not differ in interest/enjoyment, perceived competence, effort/importance, perceived choice, and value/usefulness, whether the OP was adopted or not. Interestingly, such findings conflict with the results of the open pedagogy research conducted by Clinton-Lisell and Gwozdz (2023), as they found students in the OP group presented significantly higher levels of interest/enjoyment and perceived choice. The differences in the findings may be due to the fact that their study focuses on students from a wider range of disciplines, including political science, education, mathematics, biology, business, criminal justice, social work, and English literature; however, our study focuses only on the subject of computer programming. These speculations would be supported by the previous studies that have demonstrated that the significant differences in student learning outcomes and approaches exist across different disciplines, emphasizing that findings can be contextually different and affected by disciplines (Hong et al., 2024; Meng et al., 2025; Swarat et al., 2017).

As for computational thinking skills, compared to the non-OP group, the OP group had significantly higher levels of computational thinking in all five dimensions, such as abstraction, decomposition, algorithmic thinking, evaluation, and generalization. These findings suggest that applying open pedagogy or OP is an effective way to enhance the development of minority students’ computational thinking in learning coding or programming. Minority students in the OP group presented higher levels of abstraction, decomposition, algorithmic thinking, evaluation, and generalization in computational thinking than the students in the non-OP group. In alignment with Trust et al. (2023) and Short et al. (2024), the development of skills such as critical thinking and problem solving can be enhanced through open pedagogy assignments. Specifically, the assignment of creating instructional materials for learning computer programming in the OP group was different from the common traditional coding assignment in the non-OP group. In the OP group, when the students planned the instructional content, they needed to think how to clearly explain and convey the abstract concepts of code for their audience, especially computer programming novices. This activity might encourage them to practice their ability to extract the key information and concepts from what they learned in the computer programming lectures (i.e., Variables, Operators, Control Flows, Inputs, and Methods), and to divide the complex programming content into logically simplified content that their target audience can understand. They were required to ensure that not only their instructional materials presented clear steps or well-organized content for their audience to follow but also that their explanations were effective and understandable. These viewpoints are aligned with the other studies demonstrating that engaging students in activities of designing or creating instructional materials can enhance their conceptual understanding of the target topics (Bacia, 2024; Kobayashi, 2023; Saborío-Taylor & Ramírez, 2023). Additionally, activities that position students as content creators rather than passive learners, particularly through the development of teaching-oriented materials in introductory programming courses, can promote their transferable skills to apply what they have learned in new problems (Clinton-Lisell, 2021; Saborío-Taylor & Ramírez, 2023). Through designing the examples for the designated codes in their materials by their own, the students were engaged in thinking about how to apply the code to resolve specific questions or problems. These processes could help enhance the abilities of abstraction, decomposition, algorithmic thinking, evaluation, and generalization in computational thinking. Thus, compared to the students in the non-OP group who passively only worked on resolving the coding questions given by the instructor, the students in the OP group experiencing the creation of instructional materials presented higher levels of computational thinking skills.

In terms of minority students’ perceived learning performance in computer programming, the OP group presented significantly higher levels of understanding of programming than the non-OP group. There were no significant differences in application of programming and problem-solving of programming between the OP group and the non-OP group, although the OP group showed higher averages in programming and problem-solving of programming than the non-OP group. The findings imply that the adoption of open pedagogy had a significant effect on minority students’ perceived learning performance in the aspect of understanding of programming, but not on the aspects of application and problem-solving of programming. That is, minority students’ perceived learning performance in the level of understanding of programming can be enhanced through the use of open pedagogy. This result is aligned with previous research that indicated the approach of open pedagogy facilitates the improvement of student learning performance or outcomes (Mayer, 2023; Short et al., 2024; Tlili et al., 2023). It also makes sense as minority students in the OP group had to develop the materials for others to learn coding or programming, and such development work of OER materials for coding would require students to deepen their knowledge or levels of understanding of coding/programming before thinking of ways to organize the content to be present in the learning materials for learners who are new to coding/programming (Kobayashi, 2023; Saborío-Taylor & Ramírez, 2023). On the other hand, there are several speculations about why the OP group did not show significantly higher levels of problem-solving of programming than the non-OP group. Because the OP group was asked to design the code scripting examples using OERs, they would tend to design the examples they already knew how to resolve rather than actively designing the examples that were also challenging for themselves. The non-OP group was asked to resolve the programming questions the instructor gave. That is, student-designed questions or assignments might lead to the focus on easier or more familiar materials and content as well as a potential tendency toward less challenging tasks (Doyle & Buckley, 2022; MacNaul et al., 2021; Shakurnia et al., 2018). The design of the difficulty levels of those questions or the success rates of resolving those questions might potentially affect their perceptions regarding their programming problem-solving ability. For example, if the students could not successfully resolve most of the programming questions, they might feel they had lower problem-solving ability in programming. On the other hand, success on easy or familiar questions may inflate self-assessment of problem-solving skills without reflecting a deeper understanding or transferable strategies, leading to a mismatch between perceived and actual competence (Bal & Or, 2023; Sockalingam et al., 2011; Wang, 2021).

## 6. Conclusions and Implications

This study investigated the effect of open pedagogy on minority students’ learning in computer programming, with a focus on motivation, computational thinking, and perceived learning performance in computer programming. The findings of this study not only add to the lack of existing research on the adoption of open pedagogy among minority students, but also increase the understanding of the use of open pedagogy approaches (i.e., creation and design of OERs), in comparison to the traditional approach, to enhance students’ motivation and experiences in learning programming, especially for minority students. By comparing the OP group to the non-OP group through an experimental design, minority students participating in the open pedagogy activities were found to have significantly higher motivation, computational thinking, and perceived learning performance in computer programming, compared to those in the non-OP group where traditional teaching without the use of open pedagogy was applied. Minority students in the OP group perceived significantly better performance in the understanding aspect of programming than the students in the non-OP group, but not in application and problem-solving aspects of programming in which the OP group tended to have higher averages than the non-OP group. The adoption of open pedagogy also led to the development of significantly higher computational thinking skills for minority students in all five aspects, including abstraction, decomposition, algorithmic thinking, evaluation, and generalization. From the motivational aspect, minority students with the open pedagogy instruction in learning coding presented higher levels of pressure or tension than their counterparts in the traditional non-OP instruction.

This study suggests several practical implications for educators, course instructors, instructional designers, or OER practitioners who are interested in applying open pedagogy or developing OP-integrated courses or activities for minority undergraduate students to learn coding/programming. First, when learning coding/programming through participating activates, projects, or sessions with open pedagogy, instructors should provide additional support or guidance to the students who show feelings of pressure or tension when using OER to develop materials for learning coding. Second, presenting examples of OER materials in the field of coding/programming created by others before minority students begin to use OER for the development work, may alleviate students’ levels of pressure or tension towards learning coding or programming. Third, instructors in higher education who teach minority students should consider integrating open pedagogy into courses related to coding or programming, as the use of OP has a potential to enhance minority students’ development of computational thinking, and their levels of understanding of programming. Policymakers should pay attention to the affordances of open pedagogy and its potential to enhance minority students’ successful learning experiences in computer programming education. In addition, policymakers should consider developing guidelines or guidebooks for implementing open pedagogy to facilitate educators’ adoption of open pedagogy to teach programming, and promoting the offering of professional development opportunities in open pedagogy for educators teaching coding, computer programming, or relevant subjects.

There are some limitations for this study. First, we did not control for the impact of confounding factors (e.g., previous exposure to coding or programming outside of the class, learning styles, etc.) which might have a potential impact on students’ learning experiences. Second, this study collected quantitative data and reported findings based on them, which might not fully capture students’ in-depth experiences of participating in an OP session. Third, the duration of the implementation with open pedagogy may have a potential influence on minority students’ outcomes. This study had a short implementation time of 3 weeks. Fourth, the sample sizes of 28 and 30, respectively, for the control and experimental groups, were small but sufficient in this study. Lastly, we did not conduct a pilot study before administering the survey to the students in the class, due to the constraints to reaching out to another group of students in programming courses. In addition, two different types of scales were used in the questionnaire in order to maintain the original validated structure of each instrument, which may possibly lead to a bias in number interpretations from the audience.

As for the future directions for research, it is suggested that researchers include other outcome variables to evaluate students’ learning in coding/programming, such as self-efficacy, anxiety, OER experience, technology acceptance, etc. (Y. T. Kuo & Kuo, 2023; Y. C. Kuo et al., 2023; Y. C. Kuo, 2026), which were not investigated in this study. With the quantitative analyses being the major approach to analyzing the data, it would be useful for future studies to collect some qualitative data to explore further about minority students’ perceptions towards the adoption of open pedagogy/OP in helping them learn coding or programming. In terms of the implementation time for open pedagogy, researchers may consider integrating open pedagogy/OP into a course for a longer period of time, such as a half or the full semester, in their future studies. In addition, future studies could include a larger sample size of minority students for the experimental study to verify the impact of open pedagogy on minority students’ learning in computer programming.

## Figures and Tables

**Table 1:** Background Information.

Characteristic	*n*	%
*Non-OP Group*		
Gender		
Male	15	53.6
Female	13	46.4
Age		
20	9	32.1
21	13	46.4
22	2	7.1
23	3	10.7
29	1	3.6
Ethnicity		
African American	28	100
Hispanic	0	0
Grade level		
Junior	24	83.3
Senior	4	16.7
*OP Group*		
Gender		
Male	15	50
Female	15	50
Age		
19	1	3.3
20	16	53.3
21	10	53.3
23	3	10
Ethnicity		
African American	29	96.7
Hispanic	1	3.3
Grade level		
Junior	25	83.3
Senior	5	16.7

**Table 2:** Instruments.

Scales	Number of Items	Range	Cronbach’s Alpha
Motivation	34	1–7	0.915
Interest/Enjoyment	7	1–7	0.878
Perceived Competence	6	1–7	0.894
Effort/Importance	5	1–7	0.816
Pressure/Tension	5	1–7	0.801
Perceived Choice	7	1–7	0.830
Value/Usefulness	4	1–7	0.958
Computational Thinking	19	1–5	0.946
Abstraction	4	1–5	0.890
Decomposition	3	1–5	0.816
Algorithmic Thinking	4	1–5	0.920
Evaluation	4	1–5	0.887
Generalization	4	1–5	0.851
Perceived learning performance in computer programming	33	1–5	0.982
Understanding of Programming	11	1–5	0.953
Application of Programming	11	1–5	0.947
Problem-Solving of Programming	11	1–5	0.948

**Table 3:** *t*-test Analysis for Motivation between the Non-OP Group and the OP Group.

	Groups	*n*	*M*	*S.D.*	*t*	*p*	*d*
Interest/Enjoyment	Non-OP	28	4.99	1.23	0.849	.399	0.22
	OP	30	4.71	1.25			
Perceived Competence	Non-OP	28	4.69	1.15	0.742	.474	0.20
	OP	30	4.47	1.09			
Effort/Importance	Non-OP	28	5.10	1.06	−0.872	.387	0.23
	OP	30	5.35	1.08			
Pressure/Tension	Non-OP	28	3.86	1.09	−2.188 *	.033	0.57
	OP	30	4.49	1.09			
Perceived Choice	Non-OP	28	4.29	0.82	1.632	.108	0.43
	OP	30	3.93	0.82			
Value/Usefulness	Non-OP	28	5.07	1.49	−0.809	.422	0.21
	OP	30	5.34	1.02			

*Note.* * *p* < .05; *d* refers to Cohen’s *d*.

**Table 4:** *t*-test Analysis for Computational Thinking between the Non-OP Group and the OP Group.

	Groups	*n*	*M*	*S.D.*	*t*	*p*	*d*
Abstraction	Non-OP	28	3.77	0.63	−3.769 ***	.000	0.99
	OP	30	4.34	0.52			
Decomposition	Non-OP	28	3.76	0.63	−2.131 *	.037	0.56
	OP	30	4.12	0.65			
Algorithmic Thinking	Non-OP	28	3.82	0.70	−3.115 **	.003	0.82
	OP	30	4.33	0.53			
Evaluation	Non-OP	28	3.78	0.72	−2.008 *	.049	0.53
	OP	30	4.13	0.58			
Generalization	Non-OP	28	3.86	0.59	−2.267 *	.027	0.60
	OP	30	4.22	0.61			

*Note.* * *p* < .05, ** *p* < .01, *** *p* < .001; *d* refers to Cohen’s *d*.

**Table 5:** *t*-test Analysis for Perceived Learning Performance in Computer Programming between the OP Group and the non-OP Group.

	Groups	*n*	*M*	*S.D.*	*t*	*p*	*d*
Understanding of Programming	Non-OP	28	3.50	0.87	−2.241 *	.029	0.59
	OP	30	3.94	0.60			
Application of Programming	Non-OP	28	3.45	0.87	−1.834	.072	0.49
	OP	30	3.82	0.62			
Problem-Solving of Programming	Non-OP	28	3.47	0.83	−1.843	.071	0.49
	OP	30	3.83	0.63			

*Note.* * *p* < .05; *d* refers to Cohen’s *d*.

## Data Availability

Please contact first author for information about data availability.
